# Effects of Polyphenols on Thermogenesis and Mitochondrial Biogenesis

**DOI:** 10.3390/ijms19092757

**Published:** 2018-09-13

**Authors:** Tanila Wood dos Santos, Quélita Cristina Pereira, Lucimara Teixeira, Alessandra Gambero, Josep A. Villena, Marcelo Lima Ribeiro

**Affiliations:** 1 Laboratory of Immunopharmacology and Molecular Biology, Sao Francisco University Medical School, Av Sao Francisco de Assis, 218, Braganca Paulista, São Paulo 12916-900, Brazil; quelitapereirapa@gmail.com (Q.C.P.); lucimara.teixeira@usf.edu.br (L.T.); alessandra.gambero@usf.edu.br (A.G.); 2 Programa de Pos Graduação em Genetica e Biologia Molecular, State University of Campinas, Campinas, São Paulo 13083-881, Brazil; 3 Laboratory of Metabolism and Obesity, Vall d’Hebron-Institut de Recerca, 08035 Barcelona, Spain; josep.villena@vhir.org

**Keywords:** polyphenols, mitochondrial biogenesis, thermogenesis

## Abstract

Obesity is a health problem worldwide, and energy imbalance has been pointed out as one of the main factors responsible for its development. As mitochondria are a key element in energy homeostasis, the development of obesity has been strongly associated with mitochondrial imbalance. Polyphenols are the largest group of phytochemicals, widely distributed in the plant kingdom, abundant in fruits and vegetables, and have been classically described as antioxidants owing to their well-established ability to eliminate free radicals and reactive oxygen species (ROS). During the last decade, however, growing evidence reports the ability of polyphenols to perform several important biological activities in addition to their antioxidant activity. Special attention has been given to the ability of polyphenols to modulate mitochondrial processes. Thus, some polyphenols are now recognized as molecules capable of modulating pathways that regulate mitochondrial biogenesis, ATP synthesis, and thermogenesis, among others. The present review reports the main benefits of polyphenols in modulating mitochondrial processes that favor the regulation of energy expenditure and offer benefits in the management of obesity, especially thermogenesis and mitochondrial biogenesis.

## 1. Introduction

Obesity is one of the main risk factors for the development of chronic diseases and premature death and affects more than 400 million people worldwide [1]. Progression of obesity is linked to multiple factors such as environment, lifestyle, and genetic background. It is well known that obesity is strongly associated with the development of several diseases such as hypertension, diabetes, cardiovascular diseases and cancer. In particular, excessive fat accumulation in the abdominal region is recognized as one of the main factors triggering obesity-related metabolic disorders. This fact still instigates intense speculation about the distribution of body fat, and points to its pathophysiological importance in the different compartments [2,3]. Several studies have shown that obese individuals, with a higher proportion of visceral adipose tissue compared to subcutaneous adipose tissue, present a higher prevalence of diabetes, metabolic syndrome, hepatic steatosis, atherosclerosis, and dyslipidemias, which together contribute to an increase in cardiometabolic risk [4,5,6].

Energy metabolism, wherein the mitochondria play a key role, has a negative impact on body weight. Mitochondria are dynamic organelles whose main function is production of ATP through oxidative phosphorylation. They also regulate various cellular functions such as apoptosis, calcium homeostasis, and production of reactive oxygen species (ROS), among others [7]. In this sense, due to its critical roles and multiple functions, mitochondrial dysfunction is directly or indirectly implicated in the origin of numerous diseases, such as obesity and type 2 diabetes mellitus (T2D) [8]. Obesity has been shown to be associated with decreased mitochondrial respiration, increased mitochondrial ROS production, dysregulation of mitochondrial biogenesis, decreased mitophagy signaling and increased apoptosis [9].

In view of this, therapeutic strategies that aim to restore mitochondrial function could be a promising alternative in obese individuals and hence have been extensively explored. It is well established that weight loss, as well as its maintenance, through caloric restriction and physical activity are an effective and safe strategy to control obesity. In addition, pharmacological strategies of weight loss might be considered. The Food and Drug Administration (FDA)–approved drugs includes: orlistat (reducing fat absorption by inhibiting pancreatic lipase); lorcaserin (serotonin receptor agonist that acts in the brain to reduce food intake); liraglutide (glucagon-like receptor 1 agonist, which reduces food intake); diethylpropion, phentermine, phendimetrazine, and benzphetamine (noradrenergic drugs, which function as an appetite suppressant); phentermine–topiramate (appetite suppressants that increase the release of serotonin, noradrenaline and dopamine); naltrexone–bupropion (increase satiety and decrease appetite, inhibiting the reuptake of dopamine and noradrenaline, blocking μ-opioid receptor and activating pro-opiomelanocortin) [10]. However, most of them have limited efficacy in long-term treatments, as well as present serious side effects [11,12,13]. Thus, preventive therapeutic strategies provide important alternatives to the use of conventional drugs. Thus, identifying natural compounds from food, as well as the use of plants, has aroused great interest in the last decades. This is mainly because they present reduced side effects and can act on multiple pathways to control obesity [14]. In this context, polyphenols are the class of compounds which are most intensively exploited for their antiobesity potential. They are found in abundance in the plant kingdom and are classically established as antioxidant molecules. The observed antioxidant effects are attributed to their chemical characteristics and the capacity to eliminate carbon molecules, lipid radicals, and ROS [15].

The mechanisms by which natural compounds protect against obesity include the modulation of adipogenesis, lipid metabolism, secretion of adipokines, as well as an improvement in insulin sensitivity. In addition, protective effect of some natural compounds on mitochondrial function has been demonstrated [15]. The present work describes the main findings regarding the ability of polyphenols to restore mitochondrial function through the stimulation of mitochondrial biogenesis and thermogenesis, two main mechanisms involved in the modulation of energy expenditure.

## 2. Effects of Polyphenols on the Modulation of Mitochondrial Biogenesis

Considering the relevance of mitochondria in the ATP synthesis and the high energy demand of most the cells, it is evident that mitochondrial biogenesis is a particularly important process. Although mitochondrial biogenesis is regulated by a large number of co-activators and transcription factors, the peroxisome proliferator-activated receptor gamma coactivator 1 family (PGC-1) plays a fundamental regulatory role in this process. The family is composed of three members, PGC-1α, PGC-1β and PRC (PGC-1-related coactivator), which share structural features and modes of action, as well as the capacity to regulate mitochondrial biogenesis in a wide variety of tissues [16]. They stimulate the activity of nuclear respiratory factors 1 and 2 (NRF1/2) and nuclear receptors ERRs (estrogen-related receptors) the two fundamental families of factors involved in the control of mitochondrial biogenesis. In addition, PGC-1 co-activators regulate the expression of mitochondrial transcription factor A (TFAM), a key factor that regulates mitochondrial DNA (mtDNA) replication and transcription [17]. The PGC-1α activity is regulated at post-translational level by phosphorylation, methylation and acetylation. Reversible acetylation of PGC-1α significantly modifies its transcriptional activity, and is primarily controlled, both in vitro and in vivo, by Sirtuin 1 (SIRT1) [18]. Several polyphenols have demonstrated the ability to activate SIRT1 in vitro, and they are, therefore, being investigated as potential inducers of mitochondrial biogenesis through deacetylation-mediated PGC-1α activation [19].

### 2.1. Resveratrol

Resveratrol has been shown to be effective in inducing PGC-1α activity in the liver and muscle of mice by facilitating SIRT1-mediated deacetylation, which in turn activates its transcriptional activity [20,21]. Interestingly, in addition to an increase in mitochondrial mass, resveratrol improves the survival rate [20] and motor function [21] of mice fed a high fat diet. Recently, the mitochondrial biogenesic effects of resveratrol on the SIRT1/PGC-1α pathway was also observed in an in vitro model of endothelial cells [22] and in vivo, in the aorta of T2D mice, as well as in heart of transgenic rats expressing human renin and angiotensinogen genes [23]. Although the SIRT1/PGC-1α effects are well documented, there is controversy regarding the resveratrol mode of action. Several authors have proposed that the effects of resveratrol on mitochondrial biogenesis is mediated by the activation of AMPK (AMP protein kinase) [24]. In fact, there are evidences showing that resveratrol acts primarily by activating AMPK [25], through the inhibition of phospodiesterases (PDE), ATP synthase, or OXPHOS complex III [26,27,28]. Additionally, it has been proposed that the resveratrol effect on AMPK indirectly activates SIRT1 by elevating intracellular levels of NAD^+^ [25,29]. Other authors, however, showed that resveratrol activates SIRT1 which leads to deacetylation of Liver kinase B1 (LKB1) and activation of AMPK and improve mitochondrial function both in vitro and in vivo [30,31,32].

In addition, it has been observed that resveratrol interacts directly with the oxidative phosphorylation (OXPHOS) system. Modulation of the complex I was observed on treatment with this polyphenol, mainly through its binding to subunits of Nicotinamide adenine dinucleotide (NADH) dehydrogenase, thus, competing for the Nicotinamide adenine dinucleotide (NAD^+^) binding site. This interaction, however, resulted in an antagonic effect on OXPHOS activity, where low doses of resveratrol stimulated the activity of complex I while high doses resulted in inhibitory effect on liver cells [33]. In an in vivo experiment, resveratrol significantly increased the activity of complex I in young mice, but not in old mice [34]. In vitro, resveratrol inhibits complex III activity by competing with coenzyme Q [35]. On the contrary, a recent in vitro study demonstrated an increase in the cellular respiratory capacity, as well as activities of the complexes I to IV on resveratrol treatment [36]. It was also shown that resveratrol exerts regulatory effects on the complex V and ATP synthesis, wherein low concentrations activate the complex V [37] and induce an inhibitory effect on ATP synthesis [38].

Recently, the effect of resveratrol on cells harboring mtDNA replication defects was evaluated. In human skin fibroblasts isolated from patients with homoplasmic mutations of the *Mitochondrially Encoded TRNA Leucine 1* (UUA/G)(MT-TL1), *Mitochondrially Encoded TRNA Lysine* (*MT-TK*), and *Mitochondrially Encoded ATP Synthase Membrane Subunit 6* (*MT-ATP6*) gene, treatment with resveratrol increased basal oxygen consumption rates and ATP production [39]. Resveratrol also stimulated mitochondrial fusion, resulting in larger and more highly branched mitochondrial networks. Additionally, the compound was shown to induce an increase in mitochondrial biogenesis in the skin fibroblasts from patients with deficiency in the complexes I and IV [40]. Similarly, in cells isolated from patients with complex I deficiency, treatment with resveratrol resulted in normal lactate/pyruvate ratios as well as it restored NADH/NAD^+^ ratios. This is a promising finding, since patients with OXPHOS deficiency generally present severe lactic acidosis [41] and treatment with resveratrol can prove beneficial in this condition. Reserveratrol treatment was also beneficial in fibroblasts isolated from patients with complex I deficiency. The authors found a significant reduction in ROS levels and increased levels of SOD2 mediated by an increase in SIRT3 activity [42]. Thus, the present evidences point to the beneficial effects of resveratrol in OXPHOS related diseases.

### 2.2. Quercetin

Quercetin is another polyphenol that has been extensively explored and that has shown to be very effective in inducing mitochondrial biogenesis. It has been reported to activate SIRT1 and PGC-1α, and to increase the mtDNA and cytochrome C content in both skeletal muscle and brain of mice [43]. A concomitant increase in the physical endurance of quercetin-treated animals was also observed, and such functional effect was related to the increased expression of SIRT1 and PGC-1α [43]. The effect of quercetin on mitochondrial biogenesis and endurance exercise was also studied in untrained young adult male individuals. The authors observed that quercetin not only increased mtDNA copy number, but also induced a significant increase in the physical performance [44]. Similar results were also observed in vitro, in which quercetin treatment increased the mtDNA content [45], and the expression levels of PGC-1α, NRF-1, and TFAM in a dose-dependent manner. In addition, quercetin increased the activity of OXPHOS complex IV. Thus, both in vivo and in vitro data support the notion that quercetin induces mitochondrial biogenesis through the PGC-1α/NRF-1 and TFAM signaling [46].

Alternatively, the effect of quercetin on mitochondrial biogenesis was also associated with its ability to regulate the NRF-2/HO-1/PGC-1α pathway, as well as to increase expression of the complex IV in the liver of obese mice [45,47]. Quercetin has also been shown to increase mtDNA content, expression levels of SIRT1, PGC-1α, NRF-1, and TFAM in a dose-dependent manner in the hippocampus of rats exposed to hypobaric hypoxia [48]. In addition, quercetin increased the activity of complexes II, IV, and V, as well as levels of ATP, thereby regulating OXPHOS function. Similar results were observed in the hippocampus and striatum of rodents exposed to aluminum. In this model, quercetin induced mitochondrial biogenesis through the PGC-1α/NRF-1-NRF-2-TFAM pathway and restored the mitochondrial form and number [49].

### 2.3. Hydroxytyrosol

Another polyphenol that has been extensively associated with mitochondrial biogenesis is hydroxytyrosol, present in olives (and extra virgin olive oils). In vitro studies have reported the ability of hydroxytyrosol to activate PGC-1α through deacetylation by SIRT1, which in turn induced mitochondrial biogenesis in human retinal pigment epithelial cells, ARPE-19 [50]. In a subsequent study conducted by the same group, administration of hydroxytyrosol in rats modulated the PGC-1α activity, as well as the expression of mitochondrial complexes I and II in skeletal muscle of rats subjected to ergometric exercises. An increased capacity of endurance exercise in the trained group was also observed [51]. Additionally, in contrast to inducing effect of mitochondrial biogenesis observed among moderate exercise experimental group, excessive exercise decreased the PGC-1α levels. This effect was totally reverted by administration of hydroxytyrosol [51]. Another in vitro study demonstrated the effects of this polyphenol in increasing the mitochondrial function in murine 3T3-L1 (ATCC^®^ CL-173™) adipocytes. This included an increase in the activity and protein expression of mitochondrial complexes I, II, III, and V, as well as increased oxygen consumption and decreased free fatty acids content [52]. Additionally, in vitro data in human fibroblasts also demonstrated a positive effect of hydroxytyrosol on the activation of the PGC-1α pathway. This effect was associated with increased phosphorylation of PKA and CREB, which are mechanisms involved in the regulation of OXPHOS [53]. Further, in addition to inducing PGC-1α expression, hydroxytyrosol has been shown to upregulate NRF-1 and TFAM, increase mtDNA content, and ATP synthesis in endothelial cells [54].

### 2.4. Other Polyphenols

In addition to resveratrol, quercetin and hydroxytyrosol, many other polyphenols have been shown to induce similar effects on mitochondria, in vitro. The SIRT1/PGC-1α pathway-mediated activation of mitochondrial biogenesis was observed for isoflavones (such as daidzein, genistein, and formmononetin) in rabbit proximal renal tubular cells [55], flavones (such as baicalein and wogonin) in L6 skeletal muscle cells [56], and flavan-3-ol, epigallocatechin-3-gallate in skin fibroblasts from individuals with Down’s syndrome [57]. The effectiveness of green tea on mitochondrial biogenesis was tested in vivo using rats. The authors observed an increase in the mtDNA content, as well as mRNA and protein levels of PGC-1α, complex IV, and TFAM [58]. In a human study of T2D patients with heart failure, administration of epicatechin-rich cocoa increased expressions of SIRT1 and PGC-1α which in turn stimulated mitochondrial biogenesis in skeletal muscle. Additionally, it increased the levels of porin and mitochondrial complexes I and V [59].

The beneficial effect of chronic treatment of curcumin, a turmeric-derived polyphenol, in modulating mitochondrial biogenesis has also been described. Curcumin increased PGC-1α protein expression in the brain of fast-aging accelerated senescence-8 mice (SAMP8), enhanced the mitochondrial membrane potential (MMP) and ATP levels. Additionally, it restored the mitochondrial fusion process, indicating its protective effect in diseases caused by mitochondrial dysfunction [60]. In agreement with these findings, another study demonstrated that curcumin supplementation increased levels of PGC-1α, TFAM and mitochondrial respiratory complexes, especially of the complex IV, and raised the ATP levels in the brain of APO3-mutant mice [61].

**Figure 1:**
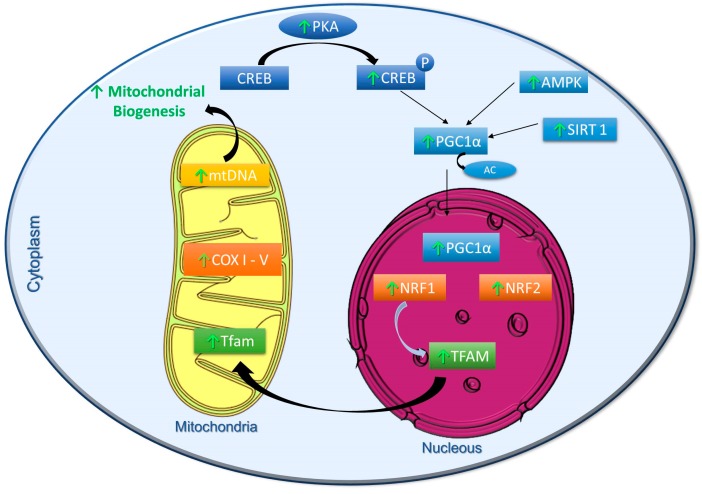
Schematic representation of metabolic pathways stimulated by polyphenols to activate mitochondrial biogenesis. PKA—Protein Kinase A; CREB—cyclic AMP response element binding protein; PGC1α—peroxisome proliferator-activated receptor gamma coactivator 1α; AMPK—AMP protein kinase; SIRT1—Sirtuin 1; NRF1/2—nuclear respiratory factors 1 and 2; mtDNA—Mitochondrial DNA; OXPHOS I-V—Oxidative phosphorylation complexes I to V; TFAM—Mitochondrial transcription factor A; P—Phosphorylation; AC—Acetylation; ↑—Polyphenol Induction.

Yerba mate (*Ilex paraguariensis*), one of the most widely consumed plant in South America, is rich in several bioactive compounds including polyphenols. A recent study conducted by our group showed that yerba mate increased mtDNA content and the mitochondrial spare respiratory capacity in cultured muscle C2C12 cells. In HFD-fed obese mice, yerba mate increased both, mtDNA content and energy expenditure in skeletal muscle and brown adipose tissue. Remarkably, these effects were associated with a decrease in body weight. Therefore, we proposed that yerba mate can partially prevent diet-induced obesity by increasing energy expenditure and enhancing mitochondrial biogenesis via the AMPK/SIRT1/PGC-1α pathway [62]. The Figure 1 summarizes the metabolic pathways stimulated by polyphenols to activate mitochondrial biogenesis.

## 3. Effects of Polyphenols on Thermogenesis

Thermogenesis is defined as the process of heat production and is generally the result of basal metabolism. Constitutive thermogenesisis sufficient to maintain body temperature of animals at thermoneutrality. However, most mammals possess a specialized type of fat tissue, called brown adipose tissue (BAT) whose function is to maintain body temperature in response to low environmental temperatures through a process termed non-shivering adaptive thermogenesis. Heat production in BAT is mediated by uncoupling protein 1 (UCP-1), a brown adipocyte-specific protein that acts as a proton channel and uncouples substrate oxidation from the synthesis of ATP, leading to dissipation of energy as heat [63]. In response to low temperatures, sympathetic pathways activate BAT through β3-adrenoreceptors [64]. In addition, the sympathetic nervous system can also be stimulated by diet, stress, and inflammation, all of which lead to an increase in thermogenesis [64,65,66]. Interestingly, together with basal metabolic rate and exercise -induced thermogenesis, diet-induced thermogenesis is one important component of daily EE [67]. In this regard, compelling evidence point towards brown adipose tissue (BAT) as a key regulatory element not only of core body temperature, but also of whole-body energy expenditure (EE). In rodents, it is believed that thermogenesis contributes to approximately 15% to 20% of total daily energy expenditure. Therefore, disorders in this mechanism can have a considerable impact on the energy balance and regulation of body weight [65]. Supporting this notion, numerous reports in rodent models and some studies in humans show that BAT activation possesses a protective role against obesity and diabetes [68,69].

Since it is unquestionable that BAT is also present in the adult humans, control of non-shivering adaptive thermogenesis has recently received huge attention [70,71,72,73]. In humans, brown adipocytes are inserted into the adipose tissues of the cervical, supraclavicular, para-aortic, paravertebral, and adrenal regions [70,74]. Increase in heat production in these regions was observed in Scandinavian workers, indicating an increase in BAT function in response to cold [75]. Seminal articles discussing the relationship between active BAT thermogenesis and obesity in rodents and humans supported the relevance of adaptive thermogenesis on the regulation of energy balance, making this mechanism an interesting target for the development of therapeutic strategies that aim at controlling body weight and fighting against obesity [74,76].

From this perspective, polyphenols have proven to be excellent thermogenic regulators. The thermogenic efficacy of green tea extracts was initially demonstrated in a study in which green tea capsules were given along with the three main daily meals. The authors observed a 4% increase in the energy expenditure (EE), an increase in carbohydrate and fat oxidation, and in the urinary excretion of noradrenaline (NA) [77]. Considering that flavonoids inhibit catechol-*O*-methyl transferase (COMT), an enzyme that degrades NA, it was suggested that green tea might interact synergistically with the sympathetic nervous system (SNS), culminating in the increase of NA-induced thermogenesis and fat oxidation [78,79,80]. In fact, this mechanism was observed by means of microcalorimetry, which demonstrated the synergistic thermogenic interactions between the sympathetic neural release of NA, caffeine, and epigallocatechingallate (EGCG) [81]. Further evidence that caffeine and EGCG might interact to increase the EE was also observed in a human study. An increase in the EE in response to the consumption of different doses of caffeine, compared with green or oolong tea products was observed. Further, regression analysis suggested that the intake of caffeine-containing products associated with EGCG promotes an even greater increase in the EE, when compared to caffeine alone [77,82,83,84]. In addition, another study demonstrated a dose-response thermogenic effect of a mixture of EGCG and caffeine on 24 h EE [83]. It has also been described that green tea catechins retain the ability to increase the EE and fat oxidation when administered even in the absence of caffeine. Thus, a caffeine-free green tea extract has been shown to increase fat oxidation during moderate-intensity exercise stress in healthy young men [85]. Interestingly, the intake of a commercially available EGCG supplement was effective in increasing the postprandial fat oxidation [86], but no increase in the resting EE was observed [86,87]. Taken together, these data suggest the possibility that, in humans, the thermogenic effects of green tea reside in the synergistic interactions between catechins, caffeine and sympathetic release of NA [81]. Additionally, it has been observed that green tea products containing caffeine and catechins reduced the respiratory quotient (indicative of increased fat oxidation) [77,82,83,88]. The thermogenic effects of green tea might be, at least in part, related with the increase in fat oxidation, which contributes to both, the effectiveness on abdominal fat loss observed in clinical trials [89,90], and weight loss maintenance [91]. Still, the unequivocal involvement of BAT in the thermogenic effects of these compounds observed in above mentioned studies still needs to be demonstrated.

Over the last few years, a wide variety of polyphenols have been investigated and their efficacy in inducing thermogenesis and fatty acids oxidation has also been demonstrated. A preparation of insoluble dietary fiber, rich in polyphenols isolated from carob, has been shown to increase postprandial EE and reduce the respiratory quotient [92]. Resveratrol and quercetin have also shown to have a thermogenic effect. Treatment with resveratrol resulted in an increase in mtDNA copy number along with an enhancement in the EE [20]. In addition, it has been shown that resveratrol supplementation reduced body fat, as well as increased thermogenesis, by increasing mitochondrial mass, and expressions of UCP1 and PGC-1α [21,93]. A similar effect was reported in another study, in which the authors observed an increase in UCP1 protein expression after reseveratrol treatment [94]. In these studies, the changes observed in UCP1 expression in adipose tissues strongly that the effects of these compounds on EE are the results of non-shivering thermogenesis activation in brown adipocytes. Quercetin supplementation led to an increase in mitochondrial density [44], and increased the endurance without previous training [95]. Reports about the efficacy of these polyphenols on metabolic rate, substrate oxidation and energy expenditure in humans are still scarce, but it is a promising field of investigation, considering the beneficial effects of grape extracts (rich in resveratrol and quercetin) in preventing obesity [96], and in reducing weight gain [97]. Studies with oleuropein, a phenolic compound found in extra virgin olive oil, have been shown to increase catecholamine secretion and increase UCP1 expression [98]. Additionally, the lemon polyphenol (hesperidin) suppressed the diet-induced obesity associated with an induction of genes related with lipid oxidation [99].

**Figure 2:**
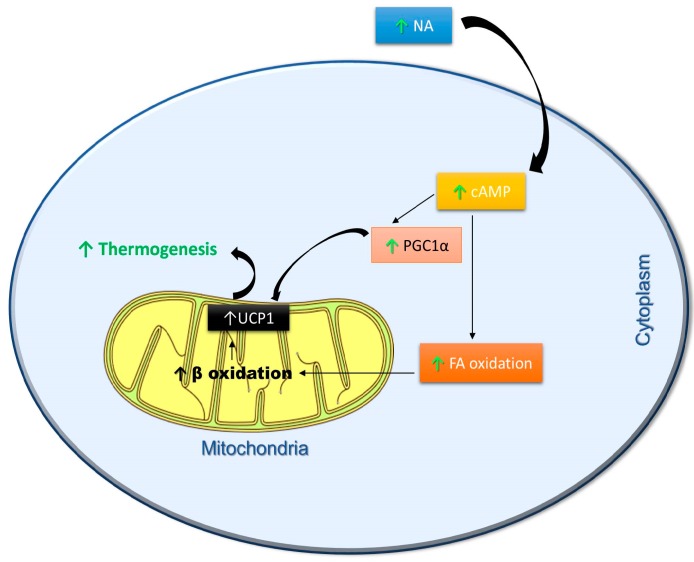
Schematic representation of metabolic pathways stimulated by polyphenols to activate thermogenesis. NA—noradrenaline; cAMP—cyclic adenosine monophosphate; PGC1α—peroxisome proliferator-activated receptor gamma coactivator 1α; FA—Fatty acid oxidation; β oxidation—Beta oxidation; UCP-1—Uncoupling protein 1; ↑—Polyphenol Induction.

Supplementation of polyphenols, such as curcumin and chlorogenic acid, led to a reduction in body weight and adiposity, apparently without a decrease in food intake, thus increasing the possibility that the compounds might express their anti-obesity effects through an EE stimulation [100,101]. Likewise, decrease in accumulation of body fat by dietary supplementation of soy isoflavones have been described [102]. Similar benefits were observed with daidzein, wherein its treatment led to weight reduction by increasing UCP1 expression [103]. In addition, it has been shown that dietary phytoestrogens help to maintain a lean phenotype, and this activity is attributed to an increased EE associated with a marked oxidation of fatty acids [104]. Finally, kaempferol, a flavonol (found in broccoli, spinach, berries), has been shown to increase cellular EE, as well as activate thyroid hormone through the stimulation of type 2 deiodinase activity [105]. Based on these findings, there is a strong possibility that the use of polyphenols to enhance UCP1-dependent and independent thermogenesis will be relevant to counteract obesity in the coming years. This is reinforced by a report from a longitudinal cohort study conducted over a 14-year period in the Netherlands, suggesting that increased dietary flavonoid intake is associated with lower weight gain in women [106]. Figure 2 summarizes the metabolic pathways stimulated by polyphenols to metabolic pathways stimulated by polyphenols to activate thermogenesis.

## 4. Concluding Remarks

The information summarized in this review clearly suggests that mitochondria represent an important intracellular target of polyphenols. Until recently, great emphasis had been placed on the antioxidant properties of these compounds, which evidently would not be limited only to their ability to eliminate ROS. Currently, it is clear that part of the antioxidant properties of polyphenols might be due to their capacity to induced mitochondrial biogenesis and improve mitochondrial function, which increases the mitochondrial efficiency leading to reduction in ROS production.

The use of polyphenols has recently been proposed as a strategy for weight loss, as they can increase EE and fat oxidation, thereby promoting weight loss. These functional compounds have the potential to produce significant effects on metabolic targets, such as activation of thermogenesis and oxidation of fatty acids. It is evident that daily increase in thermogenesis can lead to substantial weight loss, which gives polyphenols an important functional property for the prevention and treatment of a positive energy balance and obesity.
